# Immune predisposition drives susceptibility to pneumococcal pneumonia after mild influenza A virus infection in mice

**DOI:** 10.3389/fimmu.2023.1272920

**Published:** 2023-09-13

**Authors:** Sunil Palani, Md Bashir Uddin, Michael McKelvey, Shengjun Shao, Keer Sun

**Affiliations:** ^1^ Department of Microbiology and Immunology, University of Texas Medical Branch, Galveston, TX, United States; ^2^ Department of Experimental Pathology, University of Texas Medical Branch, Galveston, TX, United States

**Keywords:** influenza, *Streptococcus pneumoniae*, coinfection, genetic predisposition, pneumonia

## Abstract

**Introduction:**

A frequent sequela of influenza A virus (IAV) infection is secondary bacterial pneumonia. Therefore, it is clinically important to understand the genetic predisposition to IAV and bacterial coinfection.

**Methods:**

BALB/c and C57BL/6 (B6) mice were infected with high or low-pathogenic IAV and *Streptococcus pneumoniae* (*SPn*). The contribution of cellular and molecular immune factors to the resistance/susceptibility of BALB/c and B6 mice were dissected in nonlethal and lethal IAV/*SPn* coinfection models.

**Results:**

Low-virulent IAV X31 (H3N2) rendered B6 mice extremely susceptible to SPn superinfection, while BALB/c mice remained unaffected. X31 infection alone barely induces IFN-γresponse in two strains of mice; however, SPn superinfection significantly enhances IFN-γ production in the susceptible B6 mice. As a result, IFN-γ signaling inhibits neutrophil recruitment and bacterial clearance, leading to lethal X31/*SPn* coinfection in B6 mice. Conversely, the diminished IFN-γ and competent neutrophil responses enable BALB/c mice highly resistant to X31/SPn coinfection.

**Discussion:**

The results establish that type 1 immune predisposition plays a key role in lethal susceptibility of B6 mice to pneumococcal pneumonia after mild IAV infection.

## Introduction

Secondary bacterial pneumonia is common in influenza pandemics and epidemics, which is known to cause severe morbidity and mortality in humans. Influenza A virus (IAV) primarily targets epithelial cells, which can directly cause epithelial damage, thereby promoting bacterial colonization and invasion (1–3). Many recent studies have demonstrated that IAV-induced immune responses impair lung innate antibacterial immunity, which is a key mechanism for host susceptibility to bacterial pneumonia (4–12). Conversely, it is still unknown whether genetic predispositions contribute to IAV/bacterial co-pathogenesis.

BALB/c and C57BL/6 (B6) mice are commonly used to study the pathogenesis of viral or bacterial infections. Although wild-type (WT) mice are considered “immune competent”, there are substantial variations between two strains in their genetic predisposition. These genetic variations can affect host susceptibility to infections by regulating the efficiency of immune responses. As such, B6 mice are known to have a stronger T helper type (Th) 1 cytokine response upon infection, by producing IFN-γ, while BALB/c mice have a genetic predisposition for type 2 cytokine responses (13, 14). Nonetheless, it has been shown that the relative susceptibility of B6 and BALB/c mice to IAV infection varies depending on the viral strain and inoculum used in the infectious model (15, 16).

*Streptococcus pneumoniae* (*SPn*) is an opportunistic pathogen in humans that takes advantage of hosts with compromised immune status. Innate clearance of *SPn* is biphasic, with an early phase mediated by alveolar macrophages (AM) and a later stage dependent on neutrophil response. Thus, the relative contribution of AM and neutrophils to bacterial control depends on the inoculum used in mouse studies. AM are sufficient for clearance of low doses of *SPn* infection, while neutrophils play a key role when AM-mediated bacterial clearance is overwhelmed (17). B6 mice appear to be more efficient in AM-mediated bacterial clearance (18), whereas BALB/c mice exhibited more potent neutrophil response to *SPn* infection than B6 mice (19, 20).

The murine model of IAV/*SPn* coinfection is well-established and replicates key characteristics of human patients. We have shown that prior infection with high-virulent A/Puerto Rico/8/1934 (PR8, H1N1) in B6 mice suppresses AM function and therefore impairs acute bacterial clearance (7, 21). Additionally, PR8 infection induces AM depletion in BALB/c mice (22). Multiple studies have also shown that PR8 infection suppresses neutrophil recruitment and thereby increases susceptibility to secondary bacterial infection (9–11, 23). However, neutrophilic inflammation can also lead to acute lung damage and thereby exacerbate the coinfection progression (24, 25).

In the present study, we investigated the pathogenicity of low-virulent IAV X31 (H3N2) and *SPn* coinfection in BALB/c and B6 mice to determine the potential effects of genetic predisposition. The two groups were largely similar in their susceptibility to single infection of X31 or *S. pneumoniae* based on the strains and doses used in this study. In contrast, prior infection with a low dose of X31 induced extreme susceptibility to *SPn* superinfection in B6 but not BALB/c mice. The resistant BALB/c mice require neutrophils for effective bacterial clearance. Interestingly, B6 mice exhibited more neutrophil infiltration than BALB/c animals throughout the coinfection process, yet the bacterial control remained defective. These results suggest that type 1 immune predisposition plays a key role in inhibiting phagocyte function and therefore increasing susceptibility to pneumococcal pneumonia after mild influenza infection.

## Materials and methods

### Murine model of viral and bacterial infection

Specific pathogen-free, BALB/c WT, *Rag2* ^-/-^ *Il2rg* ^-/-^, and h*Csf2/Il3* KI mice, and C57BL/6 WT, *Ifngr1^-/-^* and *Csf2rb^-/-^* mice were purchased from the Jackson Laboratory (Bar Harbor, ME) and bred at University of Texas Medical Branch (UTMB) following Animal Care and Use Committee (IACUC) guidelines. All animal experiments were approved by UTMB, and all experiments were carried out in accordance with UTMB Assurance of Compliance with PHS Policy on Humane Care and Use of Laboratory Animals, which is on file with the Office of Protection from Research Risks, NIH.

Viral challenge was performed with a low dose (0.01 LD_50_) of X31 (~5×10^3^ PFU/mouse) or PR8 (~10 PFU/mouse, ~0.02 LD_50_) administered intranasally (i.n.) to anesthetized mice in 50 µl of sterile PBS. Titers of virus stocks and viral levels in the bronchoalveolar lavage fluids (BALF) and lungs of infected mice were determined by plaque assays on MDCK cell monolayers.

To induce bacterial pneumonia, anesthetized mice were inoculated i.n. with 50 μl of PBS containing 2×10^4^ CFU of serotype 14 strain TJO983 or 1×10^5^ CFU of serotype 2 strain D39 (26). Bacterial burdens in the BALF and lungs were measured by sacrificing infected mice at the indicated time points, and plating serial 10-fold dilutions of each sample onto blood agar plates.

### BALF cell analysis

BALF samples were collected by making an incision in the trachea and lavaging the lung twice with 0.8 ml PBS, pH 7.4. Total leukocyte counts were determined using a hemacytometer.

For flow cytometric analysis, BALF cells were incubated with 2.4G2 mAb against FcγRII/III, and stained with BV510- or FITC-conjugated anti-CD45, Allophycocyanin (APC)-conjugated anti-CD11c, APC-Cy7-conjugated anti-MHC II (I-A/I-E), BV510- or PE-Cy7-conjugated anti-CD11b, PE-Cy7- or FITC-conjugated anti-Ly6G (clone 1A8), PerCp-Cy5.5-conjugated anti-Ly6C, BV421-conjugated anti-Siglec-F, and PE- or PerCp-Cy5.5-conjugated anti-TCRβ mAbs ((H57-59, BioLegend) for myeloid cell analysis. The stained cells were analyzed on a MACSQuant analyzer. Data analysis was performed with FlowJo software.

### Determination of cytokine/chemokine production by ELISA

BALF were harvested and assayed for TNF-α, IL-1β, IL-6, IFN-γ, MCP-1 and KC by ELISA using commercially available kits from BD Biosciences and R&D Systems (Minneapolis, MN).

### Neutrophil depletion

Neutrophils were depleted using anti-Gr1 mAb RB6-8C5 or anti-Ly6G mAb 1A8 (BioXCell). Specifically, starting at one day before bacterial infection, mice were injected intraperitoneally (i.p.) with anti-Gr1 or anti-Ly6G mAb (0.1 mg/day) to deplete neutrophils or with rat IgG as a control. The efficiency of neutrophil depletion in bacterial-infected mice was confirmed by flow cytometry.

### T cell depletion

For T cell depletion, BALB/c WT mice were injected i.p. with 20 μg/mouse of hamster anti-murine CD3e mAb (145-2C11, BioXCell) every 5 days beginning 10 days before X31 infection, and then 100 μg/mouse every three days following PR8 infection. Control mice were treated with hamster IgG (HIgG). The efficiency of T cell depletion was confirmed by flow cytometry analysis of TCRβ^+^ cells.

### Statistics

Significant differences between experimental groups were determined using a two-tailed Student *t*-test (to compare two samples), an ANOVA analysis followed by Tukey’s multiple comparisons test (to compare multiple samples) or Mann Whitney test (nonparametric test) in GraphPad Prism 6 (La Jolla, CA). Survival analyses were performed using the log-rank test. For all analyses, a *P* value <0.05 was considered to be significant.

## Results

### Prior infection with low-virulent IAV induces susceptibility to pneumococcal infection in B6 but not BALB/c mice

**Figure 1:**
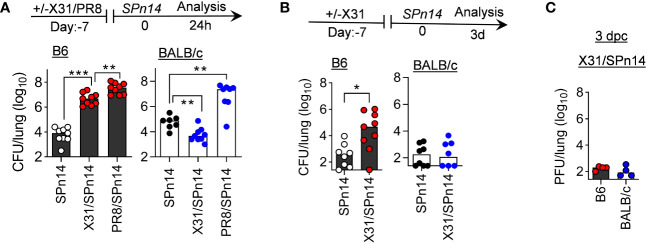
Prior infection with IAV X31 induces susceptibility to pneumococcal infection in B6 but not BALB/c mice. **(A)** Lung bacterial burdens at day 1, and **(B)** lung bacterial burden and **(C)** viral titers at day 3 after 2×10^4^ CFU *SPn14* infection of naïve, and 7-day 0.01LD_50_ X31 or 0.02LD_50_ PR8-infected B6 and BALB/c WT mice. *P< 0.05, **P< 0.01, ***P< 0.001, ANOVA analysis followed by Tukey’s multiple comparisons test **(A)** or Mann-Whitney test **(B)**. Data shown were combined from two independent experiments.

To determine whether host genetic differences contribute to the risk of secondary bacterial pneumonia, we performed side-by-side comparisons of B6 and BALB/c WT mice in their susceptibility to post-IAV *SPn* infection. Specifically, B6 and BALB/c WT mice were infected either with low-virulent X31 or high-virulent PR8 and seven days later super-challenged with a *SPn* serotype 14 strain TJO983 (*SPn14*). A low dose of X31 (0.01 LD_50_) or PR8 (0.02 LD_50_) infection alone did not induce apparent weight loss in B6 or BALB/c WT mice. However, at 7 days after PR8 infection, both B6 and BALB/c WT mice were highly susceptible to *SPn14* superinfection (**Figure 1A**). Compared with PR8/*SPn14* coinfection, B6 mice exhibited reduced bacterial outgrowth after X31/*SPn14* coinfection. Nonetheless, X31/*SPn14* coinfection resulted in ~10^3^-fold increased bacterial burdens in B6 lungs as compared with *SPn14* infection alone. In sharp contrast, prior X31 infection in BALB/c mice did not impair lung bacterial control. In fact, lung bacterial burden was significantly reduced in X31/*SPn14* coinfected BALB/c mice compared with *SPn14* infection alone. There were barely any detectable bacterial and viral burdens in BALB/c mice at 3 days post-coinfection (dpc), *i.e*, 10 days after X31 infection (**Figures 1B, C**). These striking differences between B6 and BALB/c mice suggest that host genetic traits play a significant role in susceptibility to secondary pneumococcal pneumonia after mild X31 infection.

### PR8-induced T cell activation drives susceptibility to pneumococcal infection in BALB/c mice

**Figure 2:**
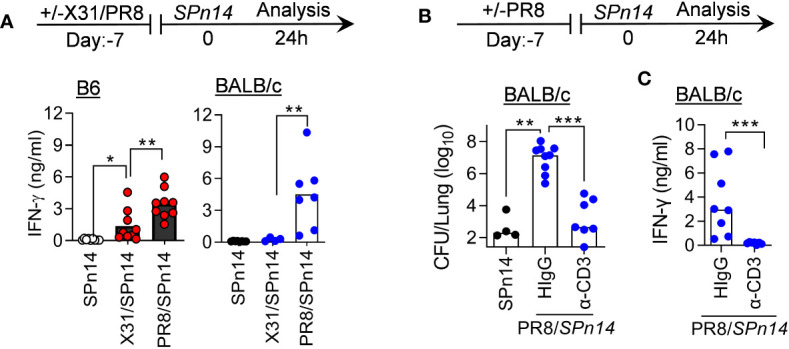
T cell activation drives susceptibility to PR8/*SPn* coinfection in BALB/c mice. **(A)** IFN-γ levels at 24 h after 2×10^4^ CFU *SPn14* infection of naïve, and 7-day 0.01LD_50_ X31 or 0.02LD_50_ PR8-infected B6 and BALB/c WT mice. **(B)** Lung bacterial burdens, and **(C)** BALF IFN-γ levels at 24 h after 0.02LD_50_ PR8 and 2×10^4^ CFU *SPn14* coinfection of BALB/c WT mice. Mice were treated with anti-CD3e mAb. Control mice were administered with hamster IgG. *P< 0.05, **P< 0.01, ***P< 0.001, ANOVA analysis followed by Mann-Whitney test. Data shown are representative of two independent experiments.

We have previously shown in both B6 and BALB/c mouse models that IFN-γ inhibits innate antibacterial immunity during recovery from PR8 infection (7, 21). In fact, PR8 infection alone is sufficient to induce prominent IFN-γ production by T cells (27). Indeed, PR8/*SPn14* coinfection resulted in prominent IFN-γ response in B6 and BALB/c mice, in agreement with their high susceptibility (**Figure 2A**). In contrast, X31/*SPn14* coinfection induced significant IFN-γ production in the susceptible B6 but not resistant BALB/c mice, indicating that IAV-induced susceptibility to *SPn14* superinfection is correlated with IFN-γ response. We next determined whether T cells are responsible for IFN-γ production in BALB/c mice after PR8 infection. T cell depletion with anti-CD3 antibodies (28) diminished IFN-γ response and completely restored initial bacterial clearance in BALB/c mice 24 h after PR8/*SPn14* coinfection (**Figures 2B, C**). It suggests that like that in B6 mice (21), PR8-activated T cells are responsible for driving susceptibility to secondary pneumococcal infection in BALB/c mice.

### Prior X31 infection does not impair innate antibacterial clearance in BALB/c mice

**Figure 3:**
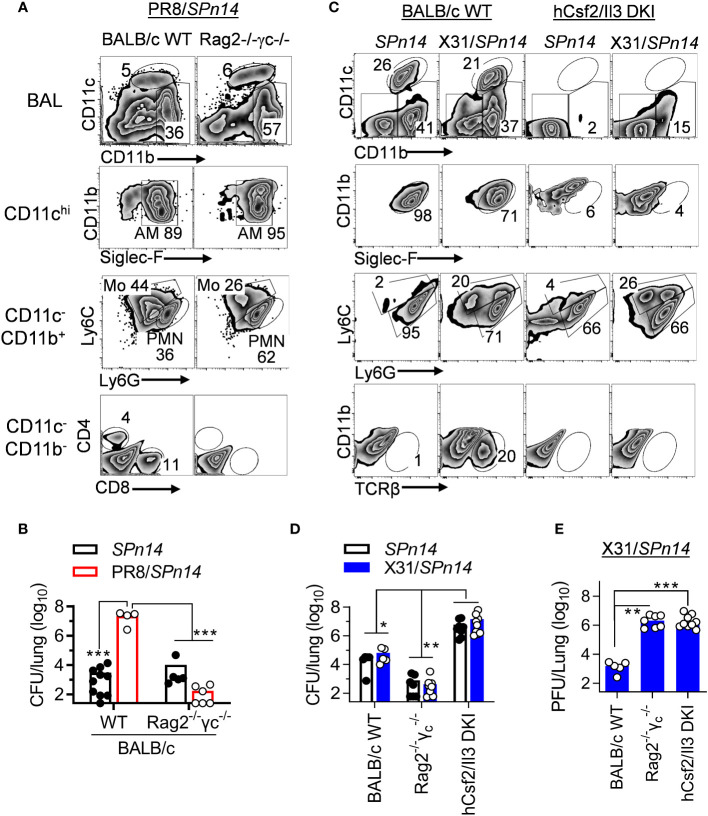
IAV X31-induced immune activation does not impair innate bacterial clearance in BALB/c mice. **(A)** Flow cytometry analysis of BALF cells and **(B)** lung bacterial burdens 24 h after 0.02LD_50_ PR8 and 2×10^4^ CFU *SPn14* coinfection of BALB/c WT and *Rag2* ^-/-^ *Il2rg* ^-/-^ mice. **(C)** Flow cytometry analysis of BALF cells, and **(D)** lung bacterial and **(E)** viral burdens 24 h after 0.01LD_50_ X31 and 2×10^4^ CFU *SPn14* coinfection of BALB/c WT, *Rag2* ^-/-^ *Il2rg* ^-/-^ and *hCsf2*/*Il3* DKI mice. *P< 0.05, **P< 0.01, ***P< 0.001, ANOVA analysis followed by Dunn’s multiple comparisons test. Data shown are representative of two independent experiments.

Phagocytes, particularly AM and neutrophils, are essential for acute clearance of pneumococcal infection. Our previous studies in B6 mice have shown that T cell-derived IFN-γ impairs AM antibacterial function (21). Thus, we wanted to examine whether this is also the case for the BALB/c mice. *Rag2* ^-/-^ *Il2rg* ^-/-^ (also known as *Rag2* ^-/-^ *γc* ^-/-^) mice are deficient in T, B and innate lymphoid cells. In the absence of IAV infection, both BALB/c WT and *Rag2* ^-/-^ *Il2rg* ^-/-^ mice were capable of efficient *SPn14* clearance (**Figures 3A, B**). Conversely, prior PR8 infection impaired acute bacterial clearance in BALB/c WT but not *Rag2* ^-/-^ *Il2rg* ^-/-^ mice (**Figure 3B**). These results agree with findings that PR8-induced T cell activation impairs innate antibacterial immunity in the lung.

Due to knock-in (KI) replacement of mouse *csf2* gene in *Rag2* ^-/-^ *Il2rg* ^-/-^ mice, human (h) *Csf2Il3* double KI (DKI) mice (29) are also devoid of AM (**Figure 3C**). *hCsf2/Il3* DKI mice exhibited ~10^3^-fold increased lung CFUs 24 h after *SPn14* infection alone, as compared with *Rag*2^-/-^ *Il2rg* ^-/-^ controls. These results indicate that AM are also required for acute clearance of *SPn14* in BALB/c mice. Note that after X31/*SPn14* coinfection, *hCsf2/Il3* DKI mice exhibited substantial monocyte and neutrophil recruitment. Importantly, prior X31 infection had no additional impact on lung bacterial control in BALB/c WT, T cell-deficient *Rag*2^-/-^ *Il2rg* ^-/-^, or AM-deficient *hCsf2/Il3* DKI mice, despite defective viral clearance in the latter two groups (**Figures 3D, E**). These findings, particularly the similar bacterial CFUs in *hCsf2/Il3* DKI mice after *SPn14* single- or super-infection, suggest that prior X31 infection has no significant impact on the AM-independent bacterial clearance mechanisms in BALB/c *hCsf2/Il3* DKI mice.

### X31-infected B6 and BALB/c mice differ in immune response to invasive *SPn* superinfection

In the absence of IAV infection, *SPn14* is innocuous to mice even after a high dose of systemic infection (7, 26). In line with that, we have shown that although X31/*SPn14* coinfection leads to lung bacterial outgrowth in B6 WT mice, it does not have significant impact on animal survival (30). We wanted to determine whether the resistance of BALB/c mice is limited to a low dose (2×10^4^ CFU) of *SPn14* superinfection. Accordingly, we used a higher dose (1×10^5^ CFU/mouse) of bacteremia strain D39 to determine the effect of genetic differences between B6 and BALB/c mice on the progression and severity of X31/*SPn* coinfection.

**Figure 4:**
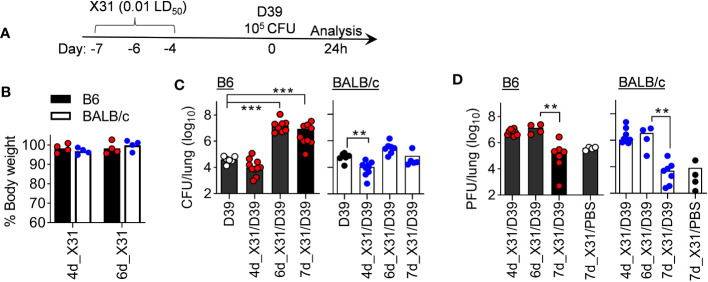
B6 mice are highly susceptibility to *SPn* superinfection 6 days after mild X31 infection. **(A)** Experimental scheme, **(B)** animal body weight, **(C)** lung bacterial and **(D)** viral burdens at various days after X31 infection of B6 and BALB/c WT mice and 24 h after infection of *SPn* D39 or PBS. **P< 0.01, ***P< 0.001, ANOVA analysis followed by Dunn’s multiple comparisons test. Data shown are representative of two independent experiments.

We first examined whether the window of susceptibility to *SPn* superinfection differs between B6 and BALB/c mice following X31 infection. Accordingly, B6 and BALB/c mice were challenged with ~10^5^ CFU of D39 bacteria at various days after X31 infection. Mice were sacrificed 24 h later for determination of lung viral and bacterial burdens, as well as cytokine responses (**Figure 4A**). Of note, a 0.01 LD_50_ dose of X31 infection alone did not induce obvious weight loss in either B6 or BALB/c mice (**Figure 4B**). Nonetheless, compared to respective D39 single-infected controls, B6 mice exhibited defective bacterial clearance starting at 6 d after X31 infection (6d_X31/D39), whereas BALB/c mice were relatively competent in their ability to clear bacteria (**Figure 4C**). Although B6 and BALB/c mice were distinct in *SPn* susceptibility at days 6 and 7 after X31 infection, D39 superinfection did not appear to interfere with lung viral clearance in two strains of mice (**Figure 4D**). Of note, both mouse strains were resistant to D39 superinfection 4 d after X31 infection (4d_X31/D39), despite high viral replication at this early time point. Lung viral titers were also comparable between two groups after 6d_X31/D39 coinfection, despite the bacterial outgrowth in B6 mice. These results verify that rather than direct viral pathogenicity, the resistance versus susceptibility of BALB/c and B6 mice to *SPn* superinfection results from their differential immune responses during recovery from IAV infection.

**Figure 5:**
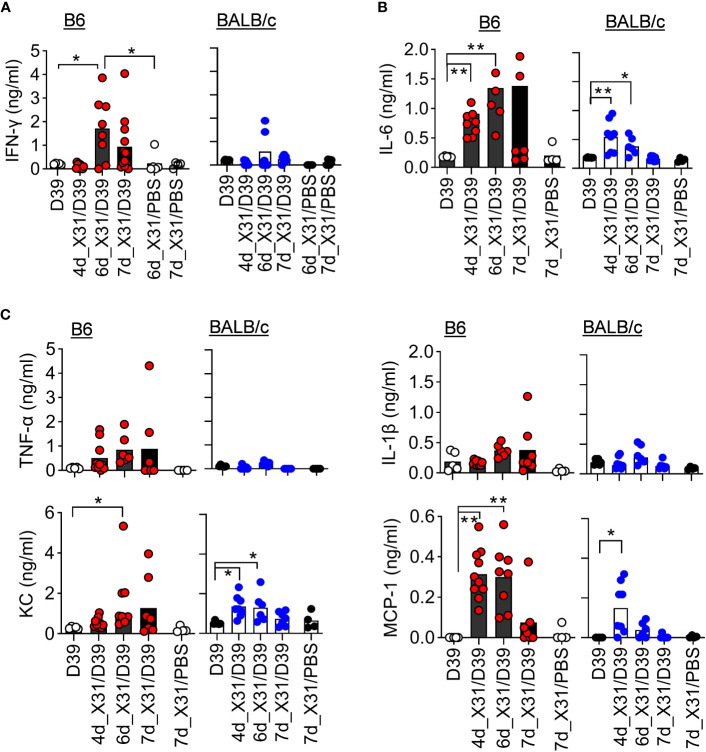
*SPn* superinfection induces differential cytokine responses in BALB/c and B6 mice after IAV X31 infection. **(A–C)** BALF cytokine and chemokine levels at various days after 0.01LD_50_ X31 infection of B6 and BALB/c WT mice and 24 h after infection of 1×10^5^ CFU D39 or PBS. *P< 0.05, **P< 0.01, ANOVA analysis followed by Dunn’s multiple comparisons test. Data shown are representative of two independent experiments.

In line with that, B6 mice exhibited significantly enhanced IFN-γ response after 6d_X31/D39 coinfection, compared with X31 or D39 single infection (**Figure 5**). Interestingly, B6 mice tended to enhance monocyte chemoattractant protein-1 (MCP-1) response, whereas BALB/c mice exhibited significantly increased neutrophil chemokine KC production after 4d_X31/D39 coinfection (**Figure 5C**). Nonetheless, D39 superinfection at this innate phase of X31 infection did not induce significant IFN-γ production in B6 or BALB/c mice (**Figure 5A**).

**Figure 6:**
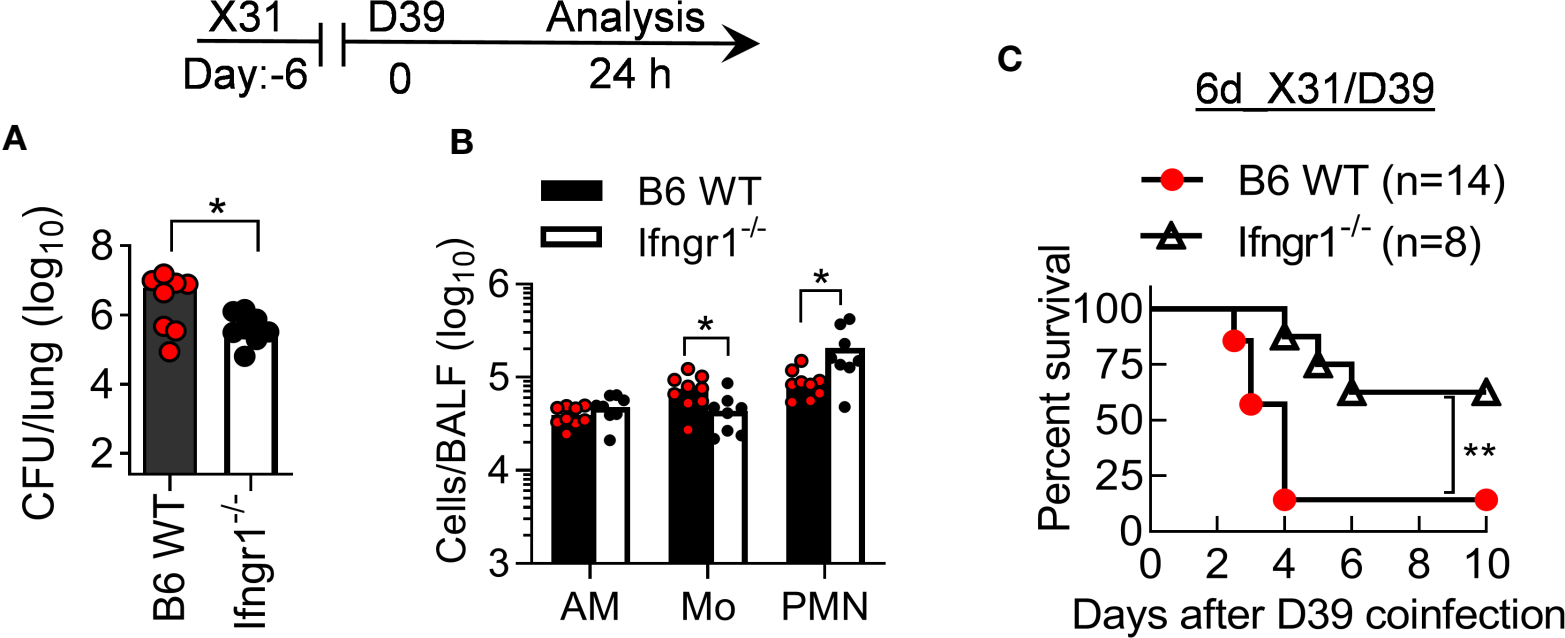
IFN-γ signaling increases the pathogenicity of X31/*SPn* coinfection in B6 mice. **(A)** Lung bacterial burdens and **(B)** BALF phagocyte numbers at 24 h, and **(C)** animal survivals after 0.01LD_50_ X31 and 1×10^5^ CFU D39 coinfection of B6 WT and *Ifngr1* ^-/-^ mice. *P<0.05, **P< 0.01, *t*-test **(A, B)** and log-rank test **(C)**. Data shown are representative of two independent experiments.

To determine the role of IFN-γ in the susceptible B6 mice, we examined the pathogenicity of X31/D39 coinfection in B6 WT and IFN-γ receptor gene-deficient (*Ifngr1* ^-/-^) mice. *Ifngr1* ^-/-^ mice exhibited increased neutrophil recruitment and bacterial clearance compared with B6 WT controls, in agreement with their significantly improved survival after 6d_X31/D39 coinfection (**Figure 6**). Thus, the susceptibility of B6 mice to *SPn* superinfection at least in part results from IFN-γ-biased immune response.

### Neutrophils are required for compensatory bacterial clearance in BALB/c mice

**Figure 7:**
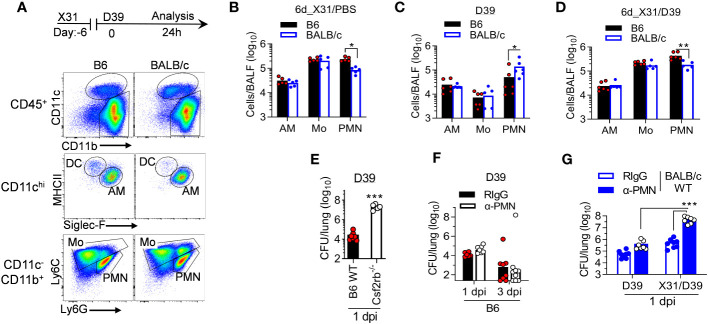
Neutrophils are required to restore bacterial clearance in BALB/c mice after X31/*SPn* coinfection. **(A)** Flow cytometry analysis, **(B–D)** BALF phagocyte numbers at 24 h after challenge of naïve, six-day 0.01LD_50_ X31-infected B6 and BALB/c WT mice with 1×10^5^ CFU D39 or PBS control. **(E)** Lung bacterial burdens 24 h after challenge of B6 WT and *Csf2rb* ^-/-^ mice with 1×10^5^ CFU D39. **(F)** Lung bacterial burdens at days 1 and 3 after 1×10^5^ CFU D39 infection of α-Gr1 (α-PMN) antibody-treated B6 WT mice. Control mice were treated with rat IgG (RIgG). **(G)** Lung bacterial burdens at 24 h after 1×10^5^ CFU D39 challenge of naïve and seven-day 0.01LD_50_ X31-infected BALB/c WT mice. Mice were treated with anti-Ly6G (α-PMN) antibody 24 h before D39 infection. Control mice were treated with rat IgG. *P<0.05, **P< 0.01, ***P< 0.001, *t*-test **(A–E)** and ANOVA analysis followed by Tukey’s multiple comparisons test **(F)**. Data shown are representative of two independent experiments.

We next analyzed myeloid cell profiles in B6 and BALB/c mice to determine whether neutrophils are responsible for their differential susceptibility to D39 superinfection (**Figure 7A**). Compared with corresponding BALB/c mice, neutrophil counts were increased in B6 mice during X31 infection alone (**Figure 7B**) but decreased with D39 single infection (**Figure 7C**). Nonetheless, B6 mice exhibited significantly increased neutrophil recruitment after 6d_X31/D39 coinfection than BALB/c mice (**Figure 7D**). Interestingly, neutrophils in BALB/c mice exhibited increased Ly6G surface expression than those in B6 animals after coinfection (**Figure 7A**). Conversely, AM numbers were comparable between two groups under different infectious conditions. Thus, it agrees with findings in the X31/*SPn14* coinfection model that rather than regulating AM or neutrophil numbers, prior X31 infection has a differential impact on the phagocyte function in BALB/c and B6 mice.

We have previously shown in B6 mice that AM are essential and sufficient for acute clearance of *SPn14* in the airway, whereas neutrophils are required for protection against *SPn14* systemic infection (7). Similarly, we found that AM were essential for acute clearance of D39 in B6 mice, as evidenced by ~10^3^-fold increased lung CFUs in *Csf2rb* ^-/-^ mice after 10^5^ CFU D39 infection alone (**Figure 7E**). On the other hand, antibody-mediated neutrophil depletion had no significant impact on bacterial control in B6 lungs (**Figure 7F**), suggesting that neutrophils are not essential for acute clearance of D39 in the absence of IAV infection. Similar to B6 mice, anti-Ly6G antibody treatment of BALB/c mice had no significant effect on acute bacterial clearance during 10^5^ CFU D39 infection alone. However, neutrophil depletion resulted in >100-fold increased bacterial burdens in BALB/c mice after X31/D39 coinfection (**Figure 7G**). These results suggest that neutrophils are essential for maintaining antibacterial immunity in BALB/c mice after X31 infection. Furthermore, the differential requirement for neutrophils before and after X31 infection indicates that the antibacterial function of AM is also impaired in BALB/c mice after X31 infection.

### BALB/c mice are resistant to otherwise lethal X31/D39 coinfection in B6 mice

**Figure 8:**
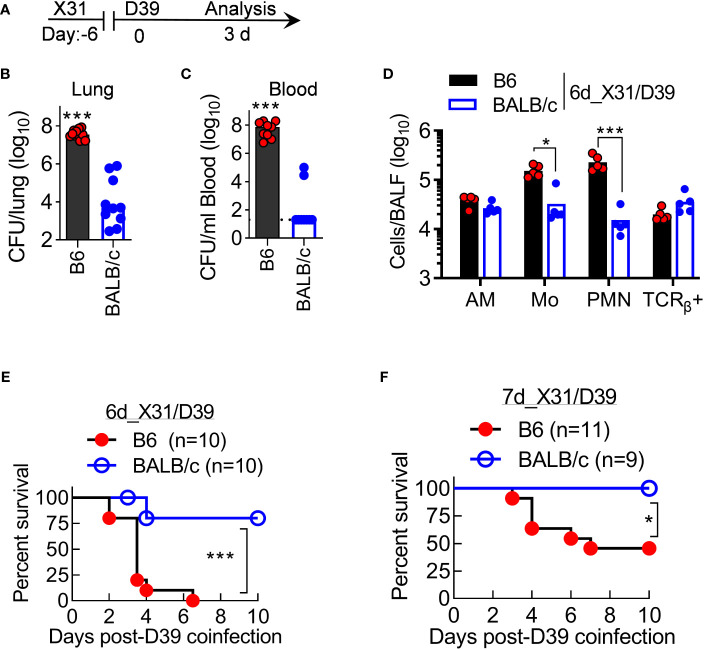
BALB/c mice are resistant to otherwise lethal X31/D39 coinfection in B6 mice. **(A)** Experimental scheme, **(B, C)** Bacterial burdens in the lungs and blood, and **(D)** BALF cell numbers at day 3 after 1×10^5^ CFU D39 challenge of six-day 0.01LD_50_ X31-infected B6 and BALB/c WT mice. Animal survival after 1×10^5^ CFU D39 superinfection at **(E)** six-day or **(F)** seven-day 0.01LD_50_ X31-infected B6 and BALB/c WT mice. *P<0.05, ***P< 0.001, *t*-test **(B–D)** and log-rank test **(E, F)**. Data shown are representative of two independent experiments.

We further investigated the progression of X31/D39 coinfection in resistant BALB/c and susceptible B6 mice. At 3 days after 6d_X31/D39 coinfection (**Figure 8A**), B6 mice not only exhibited heightened bacterial outgrowth in the lungs but also widespread bacterial invasion into the bloodstream (**Figures 8B, C**). At the same time, BALB/c mice started to resolve coinfection, as indicated by diminished bacterial burden and neutrophil accumulation in the lungs (**Figures 8B–D**). In accordance with that, B6 mice exhibited significantly increased mortality rates after X31/D39 coinfection, as compared with BALB/c animals (**Figure 8E, F**). Collectively, these data establish that antibacterial immunity is profoundly inhibited in B6 mice during recovery from X31 infection, resulting in the lethal outcome of D39 superinfection.

## Discussion

Our results demonstrate that the susceptibility to pneumococcal pneumonia after mild IAV infection is dependent on the mouse genetic background. We have shown that both BALB/c and B6 WT mice were highly susceptible to *SPn* superinfection after high-virulent PR8 infection. On the other hand, after low-virulent X31 infection, B6 mice experienced more severe *SPn* pneumonia than BALB/c mice, as evidenced by profoundly inhibited antibacterial immunity throughout the coinfection. This difference in the susceptibility was observed regardless of invasive or noninvasive *SPn* but in correlation with differential IFN-γ and neutrophil response in B6 and BALB/c mice. These findings indicate that immune predisposition plays a key role in host susceptibility to bacterial pneumonia after mild IAV infection.

Due to the clinical significance, the cellular and molecular mechanisms underlying IAV-induced susceptibility to bacterial pneumonia have been extensively investigated in animal models. These reported studies were largely accomplished using various B6 transgenic mouse models. Conversely, it remains unclear whether host genetic differences play a role in susceptibility to bacterial pneumonia, especially after subclinical IAV infection.

While both considered “immune competent,” B6 and BALB/c WT mice are distinct in immune responses to many microbial infections. It is recognized that B6 mice tend to develop Th1-biased immune response, whereas BALB/c mice are often Th2 dominant. These differences are attributable to the limited IFN-γ production in BALB/c mice (13, 14). As a result, B6 mice are more resistant to infections with intracellular pathogens such as *Leishmania major* (31) and *Yersinia enterocolitica (* 32), whereas BALB/c mice are more resistant to parasitic infection (33).

BALB/c and B6 mice are commonly used to study the pathogenicity of IAV strains. B6 mice are more resistant to highly pathogenic avian influenza A H5N1 infection than BALB/c mice, whereas BALB/c mice are more resistant to avian influenza H7N9 and pandemic H1N1 (pH1N1) infection (15, 16). Interestingly, the increased resistance of BALB/c mice to pH1N1 infection was associated with elevated IL-4 and IFN-γ production, suggesting that the Th1/Th2 cytokine paradigm is dependent on the infectious conditions.

In the present study, we infected BALB/c and C57BL/6 mice with a low dose of PR8 or X31 to study their susceptibility to *SPn* superinfection. The kinetic of viral clearance is comparable between BALB/c and B6 mice during X31 infection alone (**Figure 4D**). Compared with corresponding B6 mice, BALB/c mice exhibited increased neutrophil chemoattractant KC but decreased monocyte chemoattractant MCP-1 expression after 4d_X31/D39 coinfection. Nonetheless, both strains were resistant to D39 superinfection at this innate phase of X31 infection. We have shown in B6 mice that PR8-induced peak susceptibility to *SPn* infection coincides with lung T cell recruitment and IFN-γ expression (21). In agreement, we show here that T cell-depletion diminished IFN-γ production and restored acute bacterial clearance in PR8/*SPn*-infected BALB/c WT mice. Furthermore, BALB/c *Rag2* ^-/-^ *Il2rg* ^-/-^ mice were competent in acute bacterial clearance before and after PR8 or X31 infection, despite their deficiency in adaptive antiviral immunity.

Unlike high-virulent PR8, a low dose of X31 infection alone does not induce prominent IFN-γ production (**Figure 5A**). Interestingly, prior X31 infection enhances IFN-γ response to *SPn* superinfection in B6 but not BALB/c mice. In line with that, B6 *Ifngr1^-/-^* mice exhibited significantly improved resistance to X31/D39 coinfection, in association with increased neutrophil recruitment. Thus, IFN-γ can play multiple roles in innate suppression during IAV/*SPn* coinfection, including impairment of AM function (6, 21), inhibition of neutrophil recruitment, and likely neutrophil function. Given all these findings, it is evident that the genetic predisposition to Th1/IFN-γ-biased immune responses significantly contributes to the susceptibility to X31/*SPn* coinfection in B6 mice.

We have shown that genetic variations in AM function contribute to the susceptibility of mouse strains to pneumococcal pneumonia (18). Nonetheless, in the absence of IAV infection, AM in B6 and BALB/c mice are both competent in pneumococcal clearance. Conversely, a study by Califano et al. has shown that PR8 infection promotes IFN-γ-dependent AM depletion in BALB/c mice and thereby increases susceptibility to secondary bacterial infection, whereas IAV infection in B6 mice has no significant impact on AM survival but impairs their antibacterial function (22). In line with that, we show that BALB/c and B6 mice were similar in their susceptibility to PR8/*SPn* superinfection. On the other hand, the number of AM was similar in BALB/c versus B6 mice after X31 and/or D39 infection, and B6 *Ifngr1* ^-/-^ mice exhibited comparable AM numbers as B6 WT mice after X31/D39 coinfection. These results suggest that the differential susceptibility between B6 and BALB/c mice is not due to the impact on AM survival after X31/D39 coinfection.

Multiple studies have demonstrated that the genetic predisposition to *SPn* infection is associated with differential neutrophil recruitment (19, 34). It has been shown that after a sublethal dose of *SPn* infection alone, BALB/c mice exhibited increased neutrophil infiltration and lung tissue damage compared with B6 mice, even though their lung bacterial burden was similar (20). In agreement, we detected increased neutrophil recruitment in BALB/c mice after D39 infection alone. Due to increased neutrophil response, BALB/c mice are more resistant to the high dose of *SPn* infection than B6 mice (19). However, this differential susceptibility to *SPn* infection alone is likely dependent on bacterial strains used in the study.

Conversely, the role of neutrophils during IAV/*SPn* coinfection is controversial. It has been shown that PR8-induced type 1 IFN (IFN-I) signaling inhibits neutrophil recruitment and thereby impairs lung bacterial control in B6 mice (11). On the other hand, it is well recognized that neutrophilic inflammation is detrimental to the resolution of high-virulent IAV infection and tissue damage. Interestingly, it has been shown that BALB/c IFN-I receptor gene-deficient (*Ifnar1* ^-/-^) mice are resistant to PR8 infection like BALB/c WT animals (35), while B6 *Ifnar1* ^-/-^ mice are more susceptible than respective WT controls, due to severe neutrophilic inflammation (36). It suggests that B6 and BALB/c mice have a differential requirement for IFN-I-mediated protection against PR8 infection. Future studies are necessary to determine whether IFN-I also inhibits neutrophil recruitment in B6 mice after X31 infection. Nonetheless, we show here that compared with BALB/c mice, the susceptible B6 mice exhibited increased neutrophil accumulation throughout X31/D39 coinfection. Furthermore, we show that although dispensable during *SPn* infection alone, neutrophils are essential for bacterial clearance in BALB/c mice after X31/*SPn* coinfection. Taken together, these findings suggest that mouse genetic traits play a key role in regulating the antibacterial function of neutrophils after mild IAV infection.

Based on our findings, we propose that AM are at least partially dysfunctional in both B6 and BALB/c mice around a week after X31 infection. The resistant BALB/c mice have greater capacity to activate their neutrophils and which, in turn, contributes to the compensatory bacterial clearance, despite AM dysfunction after mild IAV infection. On the other hand, in the susceptible B6 mice, neutrophil antibacterial function is also impaired, which leads to a more severe disease course with prolonged neutrophilic inflammation and systemic bacterial invasion. The combined AM and neutrophil dysfunction eventually results in lethal X31/D39 coinfection in B6 mice. These findings provide novel information on how host immune predisposition, in addition to viral and bacterial virulence, contributes to the pathogenesis of IAV/*SPn* coinfection.

In summary, our data demonstrate that the genetic susceptibility to IAV/*SPn* coinfection is primarily attributable to the Th1/IFN-γ-biased immune response. These findings provide novel understanding of genetic risks and medical preconditions for fatal post-influenza bacterial pneumonia in humans.

## Data availability statement

The original contributions presented in the study are included in the article/supplementary material. Further inquiries can be directed to the corresponding author.

## Ethics statement

The animal study was approved by University of Texas Medical Branch (UTMB) Animal Care and Use Committee. The study was conducted in accordance with the local legislation and institutional requirements.

## Author contributions

SP: Data curation, Formal Analysis, Investigation, Methodology, Validation, Writing – original draft, Writing – review & editing. MBU: Data curation, Formal Analysis, Investigation, Methodology, Validation, Writing– original draft, Writing –review & editing. MM: Data curation, Investigation, Methodology, Writing – review & editing. SS: Methodology, Resources, Writing – review & editing. KS: Conceptualization, Formal Analysis, Funding acquisition, Project administration, Supervision, Writing – original draft.
